# Adaptive Channel Division and Subchannel Allocation for Orthogonal Frequency Division Multiple Access-Based Airborne Power Line Communication Networks

**DOI:** 10.3390/s24237644

**Published:** 2024-11-29

**Authors:** Ruowen Yan, Qiao Li, Huagang Xiong

**Affiliations:** School of Electronic Information Engineering, Beihang University, Beijing 100083, China; avionics@buaa.edu.cn (Q.L.); hgxiong@buaa.edu.cn (H.X.)

**Keywords:** adaptive algorithms, airborne communication, channel allocation, network optimization, orthogonal frequency division multiple access (OFDMA), power line communication (PLC)

## Abstract

This paper addresses the critical needs of the aviation industry in advancing towards More Electric Aircraft (MEA) by leveraging power line communication (PLC) technology, which merges data and power transmission to offer substantial reductions in aircraft system weight and cost. We introduce pioneering algorithms for channel division and subchannel allocation within Orthogonal Frequency Division Multiple Access (OFDMA)-based airborne PLC networks, aimed at optimizing network performance in key areas such as throughput, average delay, and fairness. The proposed channel division algorithm dynamically adjusts the count of subchannels to maximize Channel Division Gain (CDG), responding adeptly to fluctuations in network conditions and node density. Concurrently, the subchannel allocation algorithm employs a novel metric, the Subchannel Preference Score (SPS), which factors in both the signal quality and the current occupancy levels of each subchannel to determine their optimal allocation among nodes. This method ensures efficient resource utilization and maintains consistent network performance. Extensive simulations, conducted using the OMNeT++ simulator, have demonstrated that our adaptive algorithms significantly outperform existing methods, providing higher throughput, reduced delays, and improved fairness across the network. These advancements represent a significant leap in MAC protocol design for airborne PLC systems. The outcomes suggest that our algorithms offer a robust and adaptable solution, aligning with the rigorous demands of modern avionics and paving the way for the future integration of MEA technologies.

## 1. Introduction

Power line communication (PLC) technology leverages the existing electrical infrastructure to transmit data, providing an economically advantageous solution that significantly reduces the costs associated with network deployment and streamlines configuration processes [1,2,3]. By utilizing the ubiquitous presence of power lines, PLC eliminates the need for dedicated data cables, thus facilitating scalable network installations. This technology is extensively employed across various sectors, including in smart grids [4], where it enhances energy management and operational efficiency, and in the Internet of Things (IoT) [5], which gains from its capability to connect a vast array of devices with minimal infrastructural modifications. In particular, PLC technology is increasingly being recognized for its potential in mobile environments, such as vehicles and aircraft [6,7]. In the realm of aviation, it is instrumental in forming reliable and lightweight networks, crucial for the advancement towards More Electric Aircraft (MEA) [8,9,10,11].

Despite its advantages, the application of PLC technology in airborne environments present significant challenges. The fundamental architecture of the power distribution network, originally intended for electrical energy transmission, is not optimized for data transmission. The PLC channel is adversely affected by impulsive noise, signal attenuation, and multipath reflections, all of which arise from the complex impedance characteristics of electrical systems [12,13]. These factors collectively compromise the quality and reliability of data transmission, posing hurdles that necessitate sophisticated solutions.

Historically, research efforts in PLC have predominantly concentrated on enhancements at the physical layer to mitigate the challenges posed by the harsh channel environment. For example, ref. [12] comprehensively outlined various noise models and cancellation techniques developed to counteract interference in PLC systems, while another study [14] focused on impedance matching schemes designed to optimize transmission efficiency. Additionally, ref. [15] investigated hybrid data communication strategies that integrate PLC with wireless channels, aiming to improve both data rate and reliability at the physical layer.

On the other hand, the Medium Access Control (MAC) layer presents its own set of critical challenges that substantially impact the performance and reliability of data communication systems. Issues such as resource allocation unfairness [16], the risk of starvation [17], occurrences of collisions [18], and channel access delays [19] can severely compromise user satisfaction and the overall efficacy of the system at upper layers. Given these concerns, it is crucial to conduct a thorough examination of MAC protocols with the objective of effectively addressing these issues. Efficient MAC protocols can markedly improve system performance by optimizing channel access coordination, thereby unlocking the full potential of enhancements at the physical layer [2].

**Figure 1:**
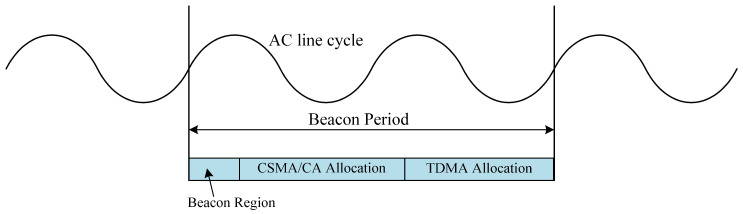
Beacon Period structure.

In typical PLC networks, a centralized architecture is employed, where a Central Coordinator (CCo) plays a pivotal role in managing node authentication and channel access. According to the widely adopted HomePlug AV2 protocol, PLC systems operate over a frequency range of 1.8 MHz to 86 MHz. These systems utilize an Orthogonal Frequency Division Multiplexing (OFDM) scheme and incorporate 4096 subcarriers. These subcarriers are organized into numerous logical subchannels to allow for simultaneous data transmissions across different channels, enhancing the network’s efficiency and bandwidth utilization. Each subchannel functions as a contention domain, and nodes desiring to utilize the same subchannel for data transmission must compete for access [20]. As depicted in Figure 1, the operational structure of these networks is organized into multiple Beacon Periods, each spanning two Alternating Current (AC) line cycles. Each Beacon Period begins with a Beacon Region, followed by distinct phases allocated for Carrier Sense Multiple Access with Collision Avoidance (CSMA/CA) and Time Division Multiple Access (TDMA) [21]. The CCo is tasked with allocating TDMA slots to traffic that requires high Quality of Service (QoS), thereby ensuring timely and reliable transmissions. In contrast, CSMA/CA is employed for less critical traffic where the risk of collision is more permissible [22]. This hybrid access mechanism optimally balances the urgent and regular data transmission demands, ensuring efficient use of channel resources and maintaining robust system performance.

Current standards in the PLC domain, such as the HomePlug AV2 protocol, along with prevailing studies on MAC layer enhancement, primarily serve applications with moderate real-time demands, typical of residential and office settings. These existing frameworks emphasize maximizing system throughput, often at the expense of other performance metrics such as latency and node fairness—metrics that are critical in airborne scenarios requiring elevated service quality. Moreover, prevailing MAC optimization techniques in PLC lack the capability to dynamically adapt the number of subchannels in response to fluctuating network conditions and node density, which often results in suboptimal performance in dynamic environments.

In response to these deficiencies, this paper introduces several algorithms aimed at meeting the unique demands of airborne PLC systems. The key contributions of our research are summarized as follows:

1. Development of an OFDMA-based multichannel CSMA/CA protocol: We have developed a novel multichannel CSMA/CA protocol tailored specifically for airborne PLC systems. This protocol significantly enhances network efficiency by optimizing throughput, minimizing delay, and ensuring equitable distribution of resources among nodes.
2. Adaptive algorithms for dynamic subchannel adjustment: Our study introduces two adaptive algorithms. The first dynamically adjusts the number of subchannels in response to fluctuations in node count and variations in network conditions. The second algorithm optimally allocates these subchannels to individual nodes, considering the quality and congestion level of each subchannel.
3. Validation through extensive simulations: The efficacy and advantages of our proposed algorithms are validated through comprehensive simulations. Results from these simulations demonstrate substantial enhancements in network throughput, reduced latency, and improved fairness among nodes.

These contributions effectively bridge the existing research gaps and provide the potential of PLC technology to operate efficiently in airborne environments with stringent real-time requirements.

The remainder of this paper is structured as follows: Section 2 reviews related work on the deployment of PLC technology in airborne applications and enhancements to the MAC layer within the framework of this technology. Section 3 delineates the system model, providing a comprehensive description to lay the groundwork for subsequent discussions. Section 4 and Section 5 elaborate on the proposed algorithms, with Section 4 detailing the channel division algorithm and Section 5 describing the subchannel allocation algorithm. Section 6 presents the results obtained from extensive simulations and provides a thorough analysis of these outcomes. The paper concludes with Section 7, summarizing the key findings and implications of this study.

## 2. Related Work

### 2.1. Empirical Validation of the HomePlug AV2 Protocol for Aviation Applications

Empirical investigations have substantiated the feasibility of the HomePlug AV2 protocol for aviation-based applications, as illustrated by studies integrating PLC within airborne Cabin Lighting Systems (CLSs) [9,10,23]. The complex topology of the power distribution networks in such systems presents a representative model for broader avionic PLC applications. Notably, the research detailed in [9] recorded a coherence time of approximately 10 ms for airborne PLC systems, which is notably longer than the duration of one Beacon Period, set at 5 ms corresponding to the 400 Hz airborne AC network. This result implies that channel conditions remain stable throughout each Beacon Period. Further studies, such as those reported in [10], have identified a coherence bandwidth of around 1 MHz at a correlation level of 0.9. Given that each subcarrier under the HomePlug AV2 standard occupies a bandwidth of 24.414 kHz, this bandwidth supports the assumption of a flat channel across each subcarrier. Additionally, the Root Mean Square Delay Spread (RMS-DS) was measured at approximately 0.1 μs, significantly less than the 10.52 μs Cyclic Prefix (CP) duration specified by HomePlug AV2, which is instrumental in mitigating inter-symbol interference.

These physical layer parameters confirm that the HomePlug AV2 protocol fulfills the essential requirements for airborne PLC systems. Building upon this empirical foundation, our research focuses on refining the MAC layer of the HomePlug AV2 protocol. The objective is to enhance its efficiency and adapt it to meet the stringent demands of real-time communication in airborne environments. This enhancement is pivotal for supporting the operational needs of contemporary and future airborne PLC systems.

### 2.2. Advancements in MAC Layer Enhancement

Recent research in MAC enhancements for OFDMA-based communication systems has introduced sophisticated approaches to address the complex challenges of diverse modern networks. In [24], the integration of target sensing with channel estimation in Terahertz massive Multiple-Input Multiple-Output systems employs a novel tensor-based parameter estimation algorithm, reducing training overhead significantly while achieving performance near the Cramer–Rao bound. This approach promotes more efficient resource sharing across the network. Concurrently, ref. [25] introduces the Distributed and Efficient Slot Assignment–Alignment Protocol (DESAA) for distributed wireless IoT networks, which significantly reduces medium access contention and message overhead by leveraging preassigned IP addresses for channel access arbitration and maintaining time slot synchronization across multihop networks. This protocol demonstrates substantial improvements in convergence time and packet loss compared to traditional carrier sense multiple access methods. Additionally, ref. [26] explores a MAC layer algorithm for hybrid communication networks combining power line and wireless media, allowing stations to compete for dual channels simultaneously, thus enhancing fairness and system throughput while reducing transmission delays.

Transitioning to PLC networks, several studies have innovated within this realm to enhance MAC performance. Ref. [27] developed a cross-layered theoretical model for IEEE 1901 power line communication networks, incorporating physical layer retransmission protocols such as ARQ and HARQ-CC to analyze their impact on MAC performance metrics and energy consumption. Similarly, ref. [28] proposed an analytical model to assess the MAC performance of the IEEE 1901 protocol in reliable PLC networks, emphasizing the influence of ARQ schemes on data transmission reliability and system throughput. In a more industrial context, ref. [29] introduced the robust preamble-based MAC mechanism (R-PMAC) for PLC networks in the Industrial Internet of Things, which enhances networking speed and supports data frame loss management through mechanisms like whitelist authentication and robust collision handling. Additionally, ref. [30] presented CodePLC, a dynamic network coding MAC protocol that uses a single relay node for broadcasting in smart grid environments, significantly improving goodput and reducing end-to-end latency. Furthermore, ref. [31] explored distributed spectrum sharing in enterprise power line communication networks, proposing a technique that enables multiple PLC links to communicate concurrently within the same collision domain, thereby enhancing aggregated and per-link throughput in HPAV PLC networks. Lastly, ref. [20] investigated resource allocation in relay-based OFDMA PLC systems, developing an optimal resource allocation scheme that maximizes system throughput by jointly optimizing subcarrier and power constraints under interference and power limitations, with the proposed scheme demonstrating efficiency and competitive performance in simulations.

In these research on the MAC layer optimization of PLC systems, one promising direction is to achieve MAC enhancement by means of the divide and allocation of subchannels. Ref. [32] explores the optimization of subchannel assignment in in-home PLC networks through a linear programming approach, aiming to maximize network throughput while ensuring traffic fairness, effectively improving performance over traditional methods. Ref. [33] presents a CSMA/CA protocol for OFDMA-based broadband PLC that leverages the stable channel characteristics of power lines to gain multiuser diversity. By dividing the entire bandwidth into several subchannels and allocating each subchannel based on channel conditions, the protocol significantly reduces the collision probability and enhances system utility. However, this approach employs a utility function defined as the logarithm of single-node throughput, which may lead to an inequitable distribution of resources. Similarly, ref. [34] introduces an opportunistic random-access scheme that assigns subchannels to users based on the variations in their channel-state in both time and frequency domains. This strategy prioritizes users with better channel conditions, optimizing system throughput in environments where the number of users substantially exceeds available subchannels. Despite its adaptability, the strategy’s fixed subchannel count could restrict its responsiveness to changes in node count and traffic density. In vehicular contexts, ref. [35] proposes a MAC protocol that combines time and frequency multiplexing for vehicular PLC systems, using a distributed channel selection policy and collision resolution algorithm to maximize transmission success across multiple channels. However, this protocol’s dependence on intricate, distributed decision-making and strict time synchronization might curtail its efficacy in dynamic environments.

## 3. System Model and Saturated MAC Throughput Definition

Consider an OFDMA-based airborne PLC network consisting of *N* nodes, operating under a centralized architecture managed by a CCo. Unlike cellular networks, where an Access Point (AP) facilitates communication, PLC networks support direct node-to-node interactions.

It is assumed that traffic within the network is saturated, meaning each node persistently maintains a backlog of data frames ready for transmission. Nodes are assumed to possess perfect Channel State Information (CSI), achievable through the sounding technique as stipulated in the HomePlug AV2 protocol [21]. Employing bit-loading technology, physical layer transmission rates are computed based on this CSI [36]. The network bandwidth is partitioned into *m* subchannels, each comprising a group of adjacent subcarriers.

**Figure 2:**
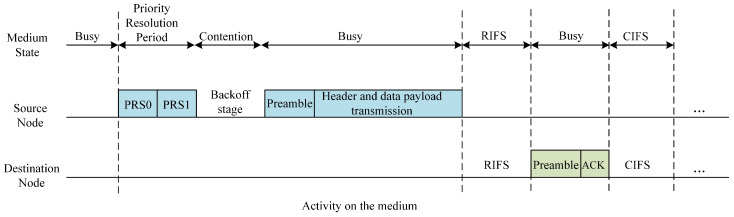
HomePlug AV2 MAC frame transmission timeline in the CSMA/CA mode.

Figure 2 illustrates the MAC frame transmission sequence under the standard CSMA/CA mode defined by the HomePlug AV2 protocol [37]. The sequence begins with a Priority Resolution Procedure (PRP), where nodes are assigned priorities across four levels using two bits, with higher-priority nodes prevailing. Collision avoidance is implemented via a random backoff mechanism. Nodes that are successful in the PRP proceed to the backoff stage, characterized by sequential, equally timed backoff slots. Here, the backoff counter (BC) for each node decrements sequentially within each slot until it reaches zero, permitting the node to transmit a preamble and thereby obtain the channel access right. Following this, the MAC frame transmission commences, comprising both header and data payload transmission.

The total transmission time, denoted as $T_{s}$, is defined by the following equation:

(1) $$T_{s}=T_{PRP}+T_{B}+T_{P}+T_{H}+T_{D}+T_{RIFS}+T_{ACK}+T_{CIFS}$$

where $T_{PRS}$, $T_{B}$, $T_{P}$, $T_{H}$, and $T_{D}$ represent the durations of PRP, random backoff, preamble, and header and data payload, respectively. $T_{RIFS}$ indicates the duration of the Response Inter-Frame Spacing (RIFS), which provides a necessary pause between the data transmission and its acknowledgement to ensure that the Acknowledge (ACK) is processed accurately. $T_{ACK}$ is the time allocated for transmitting the acknowledgment from the receiver back to the sender, confirming the successful receipt of the data. Lastly, $T_{CIFS}$ represents the Contention Inter-Frame Spacing (CIFS), which imposes a mandatory wait period to prevent immediate subsequent transmission attempts, thereby mitigating the risk of collisions on the network.

It should be noted that the calculation of $T_{s}$ does not account for the bursting and inverse bursting procedures detailed in the HomePlug AV2 protocol [37] for analytical simplicity, though this simplification maintains analytical rigor and can be extended to include these procedures. In addition, all components in Equation (1), except for the data payload transmission, constitute overhead, thus impairing the MAC layer’s efficacy in translating physical layer data rates to MAC throughput.

Under saturated traffic conditions, the aggregate duration of the header and data payload, $T_{H}+T_{D}$, is restricted by the maximum frame length, MaxFL:

(2) $$T_{H}+T_{D}=MaxFL$$

For a network with *n* nodes competing for channel access, the saturated MAC throughput, denoted as $S\left(n\right)$, quantifies the proportion of time dedicated to data transmission and is defined as follows [38]:

(3) $$S\left(n\right)=\frac{P_{tr}\left(n\right)P_{s}\left(n\right)T_{D}}{\left(1-P_{tr}\left(n\right)\right)\sigma +P_{tr}\left(n\right)\left(P_{s}\left(n\right)T_{s}+\left(1-P_{s}\left(n\right)\right)T_{c}\right)}$$

where $P_{tr}\left(n\right)$ denotes the probability of at least one node commencing transmission and $P_{s}\left(n\right)$ the probability of successful frame transmission with *n* contending nodes. σ denotes the duration of an idle slot and $T_{c}$ the time loss attributed to frame collisions caused by simultaneous preamble transmissions by multiple nodes.

## 4. Channel Division

In this section, we address the issue of channel division, specifically determining the optimal number of subchannels to achieve efficient communication within OFDMA systems.

### 4.1. Problem Formulation

When deciding the number of subchannels in an OFDMA-based airborne PLC network, a primary consideration is the overhead induced by channel division. Notably, the transmission time for header information, denoted as $T_{H}$, increases linearly with the number of subchannels due to the reduction in bandwidth per subchannel by a factor of 1/m. This reduction prolongs the duration required to transmit header details such as source and destination addresses. Mathematically, this relationship is represented as follows:

(4) $$T_{H}\left(m\right)=m\cdot T_{H}$$

Consequently, the payload transmission time is calculated as follows:

(5) $$T_{D}\left(m\right)=MaxFL-T_{H}\left(m\right)$$

For node *i*, $i\in \left\{1,\ldots ,N\right\}$, the subchannel it is assigned as $f\left(i\right)$, $f\left(i\right)\in \left\{1,\ldots ,m\right\}$. The saturated MAC throughput of subchannel $f\left(i\right)$ given in Equation (3) can then be reformulated as

(6) $$S\left(N_{f\left(i\right)},m\right)=\frac{P_{tr}\left(N_{f\left(i\right)}\right)P_{s}\left(N_{f\left(i\right)}\right)T_{D}\left(m\right)}{\left(1-P_{tr}\left(N_{f\left(i\right)}\right)\right)\sigma +P_{tr}\left(N_{f\left(i\right)}\right)\left(P_{s}\left(N_{f\left(i\right)}\right)T_{s}+\left(1-P_{s}\left(N_{f\left(i\right)}\right)\right)T_{c}\right)}$$

Equation (6) reflects the data payload transmission efficiency on a subchannel with $N_{f\left(i\right)}$ nodes assigned to it. The throughput for node *i*, denoted as $R_{i}$, is calculated as

(7) $$R_{i}=r_{i,f\left(i\right)}\frac{S\left(N_{f\left(i\right)},m\right)}{N_{f\left(i\right)}}$$

where $r_{i,f\left(i\right)}$ symbolizes the achievable physical data rate for node *i* on its assigned subchannel. By summing the throughputs of all nodes, we obtain the total system throughput:

(8) $$R=\sum_{i=1}^{N}r_{i,f\left(i\right)}\frac{S\left(N_{f\left(i\right)},m\right)}{N_{f\left(i\right)}}$$

To quantify the benefit of channel division, we introduce the Channel Division Gain (CDG), defined as

(9) $$G\left(m\right)=\frac{E\left[R\right]}{E\left[R_{single}\right]}$$

where $R_{single}$ denotes the collective throughput without channel division, and is given as follows:

(10) $$R_{single}=\sum_{i=1}^{N}r_{i}\cdot \frac{S\left(N,1\right)}{N}$$

with $r_{i}$ representing the feasible physical data rate for node *i* over the entire bandwidth when treated as a single channel.

The optimal subchannel count is the one that maximizes $G\left(m\right)$. Integrating Equations (8) and (9) into (10), $G\left(m\right)$ simplifies to

(11) Gm=m·Eri,fi·SNNmm,mEri·SN,1

Note that independence between $r_{i,f\left(i\right)}$ and SNNm,mm,m is assumed here, since the former is influenced by physical layer characteristics such as bandwidth and SNR, while the latter by MAC layer configurations.

It is obvious that SNNmm,m initially rises as *m* increases due to a decrease in collision probability with a greater count of subchannels. Subsequently, it declines as the overhead from header transmission intensifies with more subchannels.

Under the assumption of identical conditions for each subchannel for *i*, it follows that

(12) $$E\left[r_{i}\right]=m\cdot E\left[r_{i,f\left(i\right)}\right]$$

However, with varying subchannel conditions and selective subchannel allocation as elaborated in Section 5, we have

(13) $$E\left[r_{i}\right]=g\left(m\right)\cdot m\cdot E\left[r_{i,f\left(i\right)}\right]$$

where $g\left(m\right)$ ranges from 0 to 1, representing subchannel diversity. With m=1, $g\left(m\right)$ equals 1 and decreases as *m* increases until the subchannel bandwidth falls below the coherence bandwidth, after which $g\left(m\right)$ plateaus.

Substituting Equation (12) into (11) yields

(14) Gm=SNNmm,mgm·SN,1

Based on the mathematical properties of SNNmm,m and $g\left(m\right)$, we can come to the conclusion that $G\left(m\right)$ initially increases with an increasing number of subchannels, followed by a subsequent decline. In Section 4.2, we will exploit this characteristic to identify the optimum number of subchannels.

### 4.2. Algorithm Implementation

To effectively manage the computational demands associated with calculating $G\left(m\right)$ for various subchannel counts *m*, which involves extensive computations to retrieve physical data rates for each node across different subchannels and to average SNNmm,m over time, we propose an adaptive algorithm. This algorithm dynamically adjusts the optimal subchannel count in response to real-time feedback.

The algorithm initiates by setting a correlation between the subchannel count and node density, and it adapts this parameter based on changes in node count or subchannel conditions. These changes are continuously monitored by the CCo. Utilizing the properties of $G\left(m\right)$ as outlined in Section 4.1, this feedback mechanism refines channel division outcomes to optimize network performance.

Based on the coherence bandwidth parameters and the 4096 subcarriers specified in the HomePlug AV2 protocol, we establish a set of potential subchannel counts:

(15) $$M=\left\{2^{q}\right\},0\leq q\leq 7,q\in Z$$

Initially, the CCo sets the subchannel count based on node density:

(16) $$m=\min\left\{m\in M:m>N/5\right\}$$

During network operation, the CCo continuously monitors the following:

1. Node count *N*.
2. The quality discrepancy across subchannels, quantified by their variance in the signal-to-noise ratio (SNR), denoted as δ.
3. The number of successful MAC frame transmissions during the CSMA/CA phase of each Beacon Period, denoted as $n_{frame}$.

An increase in node count or a greater discrepancy in subchannel conditions typically necessitates a higher subchannel count to mitigate channel contention and to leverage subchannel diversity. Consequently, the CCo may decide to modify *m* based on changes in *N* and δ, and using $n_{frame}$ as feedback for further optimization.

To be specific, if an increase in *N* or δ persists for ten consecutive Beacon Periods, the CCo will double *m* in the subsequent Beacon Period and adjust further based on fluctuations in $n_{frame}$. According to the unimodal property of $G\left(m\right)$, which has been proven in Section 4.1, an increase in $n_{frame}$ subsequent to this adjustment suggest that *m* is within the ascending interval of $G\left(m\right)$, prompting continued doubling until no further improvement in $n_{frame}$ is observed, or *m* reaches its upper limit. Conversely, a reduction in $n_{frame}$ following an increment in *m* prompts the CCo to revert the change.

Similarly, a consistent decrease in *N* or δ over ten Beacon Periods leads the CCo to halve *m*. Adjustments continue based on the resultant changes in $n_{frame}$, with the halving process ceasing when $n_{frame}$ no longer increases, or when *m* reaches its minimum threshold. If $n_{frame}$ decreases following a reduction in m, the CCo annuls the last alternation.

Adjustments to the channels’ division are deliberately deferred until changes in channel conditions have been stably observed for ten consecutive Beacon Periods. This precaution ensures that the system does not react to transient fluctuations, thereby maintaining stability and accuracy in response. The pseudocode detailing the operational procedure of the channel division algorithm is presented in Algorithm 1.
**Algorithm 1** Adaptive optimization of subchannel count.**Input:**    1. *N*: Number of nodes in the network    2. δ: Variance in SNR of each subchannel monitored by CCo    3. $n_{\mathrm{frame}}$: Number of successful MAC frame transmissions in the CSMA/CA phase of each Beacon Period**Output:**    *m*: Optimal subchannel count**Initialization:**    Determine initial *m* based on node density from the set $M=\left\{1,2,4,8,16,32,64,128\right\}$:    $m=\min\left\{m\in M:m>N/5\right\}$**Proceure:**    Observe changes in *N* or δ persisting over ten consecutive Beacon Periods:    **if** an increase is noted **then**      Double *m*: m←2m      **if** $n_{frame}$ rises **then**        Keep doubling *m* until no further increase or $\max\left(m\right)$ is reached      **else**        Halve *m* to revert to the previous value      **end if**    **else if** a decrease is noted **then**      Halve *m*: m←m/2      **if** $n_{frame}$ ascends **then**        Persist in halving *m* until no further rise or $\min\left(m\right)$ is attained      **else**        Double *m* to cancel the last adjustment      **end if**    **end if**

### 4.3. Computational Complexity Analysis

In the channel division algorithm proposed for OFDMA-based airborne PLC networks, the CCo is tasked with continuous monitoring of the network conditions and making adaptive decisions regarding the optimal number of subchannels. The complexity of this algorithm primarily stems from the monitoring tasks and the adaptive decision-making process rather than direct computational tasks such as calculating the CDG. Instead of explicitly computing $G\left(m\right)$, the algorithm leverages known properties of $G\left(m\right)$ to guide the adjustments in the subchannel count. This approach significantly reduces the computational burden that would otherwise be associated with evaluating $G\left(m\right)$ for various subchannel configurations.

The algorithm’s efficiency is rooted in its ability to dynamically adjust subchannel allocations based on real-time feedback concerning network conditions such as node density, signal-to-noise ratio variance, and the number of successful MAC frame transmissions. The decision-making process involves a series of conditional checks which, although iterative, are computationally inexpensive, each being $O\left(1\right)$ operations. These operations are dependent on changes in network metrics, which are continuously updated and monitored; thus, the complexity associated with this part of the algorithm is primarily related to the frequency and number of these condition checks.

Overall, the computational complexity of the CCo’s tasks is largely influenced by the overhead of maintaining up-to-date network state information and the logic required to interpret this information to make timely adjustments. This process ensures the algorithm remains efficient and responsive to changes in the network environment, making it highly suitable for dynamic and resource-constrained settings such as airborne PLC systems. This methodical yet resource-efficient approach allows the system to maintain optimal performance by adapting to fluctuating network conditions without the need for intensive computations, thereby preserving the system’s capacity to handle other critical tasks concurrently.

## 5. Subchannel Allocation

Upon determining the optimal number of subchannels, the subsequent step involves their efficient allocation. This section introduces a novel subchannel allocation algorithm designed to distribute the network load effectively while considering the unique properties of each node and subchannel.

### 5.1. Metric Design

To facilitate this allocation, we establish the Subchannel Preference Score (SPS), a metric to quantify the suitability of each subchannel *j*, $j\in \left\{1,\ldots ,m\right\}$, for a given node *i*. The SPS is defined as follows:

(17) $$D_{i,j}=\frac{SNR_{i,j}}{N_{j}+1}$$

Here, $SNR_{i,j}$ denotes the SNR of node *i* on subchannel *j* and $N_{j}$ represents the number of nodes currently assigned to subchannel *j*. The inclusion of one in the denominator ensures that division by zero is avoided.

The SPS evaluates the desirability of subchannel *j* for node *i* by considering both the channel quality (SNR) and its current occupancy, providing a balance between demand and capacity.

### 5.2. Algorithm Implementation

Within the Beacon Region of each operational cycle, nodes transmit their $SNR_{i,j}$ data to the CCo for all nodes *i* and subchannel *j*. The CCo then employs a randomized selection process to choose a node, denoted as node *a*, and calculates $D_{a,j}$ for each subchannel for this node. Node a is allocated to the subchannel $J_{a}$ that offers the highest $D_{a,j}$ value, thereby optimizing the node’s performance based on the current network conditions.

Following this allocation, the occupancy of the chosen subchannel, $N_{J_{A}}$, is incremented by one to reflect the new assignment:

(18) $$N_{J_{A}}=N_{J_{A}}+1$$

This allocation cycle repeats until each node has been assigned a specific subchannel. The CCo finalizes and broadcasts these allocation decisions, which the nodes adhere to during the subsequent Beacon Period. This randomized selection method ensures long-term fairness and equitable access to the network resources over multiple Beacon Periods.

The pseudocode for this subchannel allocation algorithm is detailed in Algorithm 2.
**Algorithm 2** Subchannel allocation in OFDMA-based PLC networks.**Input:**    1. *N*: Number of nodes in the network    2. *m*: Nember of subchannels in the network    3. $SNR_{i,j}$ for each $i\in \left\{1,\ldots ,N\right\}$ and $j\in \left\{1,\ldots ,m\right\}$**Output:**    Subchannel allocation result**Initialization:**    i=0    $N_{j}=0,j\in \left\{1,\ldots ,m\right\}$**while** i<N    Randomly select a node, denoted as *a*, $a\in \left\{1,\ldots ,N\right\}$    Assign node *a* to the subchannel $J_{a}=\max_{j}\left\{D_{a,j}\right\}$, $D_{a,j}=\frac{SNR_{a,j}}{N_{j}+1}$    $N_{J_{A}}\leftarrow N_{J_{A}}+1$    i←i+1**end while**

### 5.3. Computational Complexity Analysis

The subchannel allocation algorithm detailed in this paper represents a strategic approach to managing network resources in OFDMA-based airborne PLC systems, emphasizing efficiency and fairness. The complexity of this algorithm arises primarily from the operations required to evaluate the SPS and allocate nodes based on this metric. Each operational cycle within the Beacon Region necessitates that nodes transmit their SNR data for each subchannel to the CCo, which then performs calculations to determine the most suitable subchannel for each node.

The computational burden of the algorithm is driven by the necessity to compute the SPS, $D_{i,j}$, for each node–subchannel pair. The computation of $D_{i,j}$ involves a straightforward division of the SNR by the incremented occupancy of the subchannel, ensuring division by zero is avoided. While individually these operations are not computationally intense—being $O\left(1\right)$ for each node–subchannel calculation—the complexity can accumulate over the network due to the necessity to perform these calculations for every node across potentially multiple subchannels.

During each allocation cycle, the CCo randomly selects a node, calculates the SPS for each subchannel, and then assigns the node to the subchannel with the highest score. The post-allocation adjustment of the subchannel occupancy, $N_{J_{A}}$, further adds to the computational steps, although this too is an $O\left(1\right)$ operation. The primary computational load, therefore, lies in the repeated evaluation of $D_{i,j}$ for potentially many nodes and subchannels, suggesting a complexity that could scale as $O\left(N*M\right)$, where *N* is the number of nodes and *M* is the number of subchannels.

## 6. Performance Evaluation

This section offers an in-depth evaluation of our proposed channel division and subchannel allocation algorithms designed for OFDMA-based airborne PLC networks. We conducted extensive simulations to assess performance with the open-source message-level simulator OMNeT++ [39], focusing on key metrics such as saturated throughput, average delay, and fairness among nodes. Our comparative analysis included benchmarks including the HomePlug AV2 protocol and solutions to channel contention in other academic papers.

### 6.1. Simulation Setup

**Figure 3:**
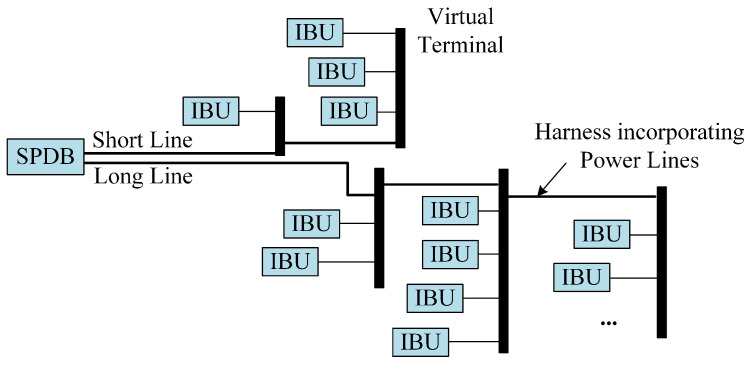
Network architecture cell schematic.

The power distribution network within the CLS, as delineated in [9], forms the basis of our simulation environment. Reflecting a typical commercial aircraft cabin, the network is organized into discrete segments, with each segment represented as a single cell. Figure 3 illustrates the schematic layout of such a cell, where the Secondary Power Distribution Box (SPDB) links all peripheral nodes through two primary lines. In our simulations, the SPDB acts as the CCo, supplying power and facilitating control message transmission to terminal nodes, specifically the Illumination Ballast Units (IBUs).

Simulations were conducted over an airborne AC power distribution network with a frequency of 400 Hz, with each AC cycle lasting 2.5 ms. To ensure the validity of our results, each simulation scenario was replicated across 100 independent runs. Simulation parameters, including the durations of CIFD, RIFS, and backoff slots, were set to 100 μs, 48.52 μs, and 35.84 μs, respectively, following the specifications outlined in [21].

The PLC channel was modeled based on extensive theoretical analysis and empirical data as a multipath channel [40], where the combined effect of multiple propagation paths results in a log-normally distributed overall channel gain [41]. Based on empirical data from [10], which include measurements of insertion gains between the SPDB and various IBUs, we established the average insertion gain at −20 dB, observed specifically between the SPDB and IBU 8. To accurately reflect these empirical findings, we adopted mean and standard deviation values for the log-normal distribution that align with the observed average gain, setting the standard deviation within the range of $\left[1,2\right]$ to account for variations in the gain distribution. The mean was adjusted correspondingly.

All nodes are assumed to operate under saturated traffic conditions with synchronized timings, and all frames are assumed with equal priority. The maximum frame length was set at 2501.12 μs, as stipulated by the HomePlug AV2 protocol.

### 6.2. Simulation Results

In this section, we conduct a comprehensive performance evaluation of the algorithms introduced in this paper, focusing on key metrics such as system throughput, average latency, and fairness among nodes. For a thorough comparative analysis, we have selected several benchmark algorithms, each distinguished by its particular operational strengths within specific communication environments:

1. The robust preamble-based MAC (R-PMAC) method proposed in [29], which is designed for robust transmission in noisy industrial settings. This method utilizes a preamble-based approach that enhances collisions management and ensures reliable frame delivery.
2. The opportunistic random-access scheme introduced in [34], which optimizes user access efficiency by dynamically adjusting backoff timings based on the real-time variances in channel-states.
3. A conventional MAC mechanism that treats the entire bandwidth as a single channel, without channel division. This serves as a baseline to highlight the advantages of implementing channel division.

**Figure 4:**
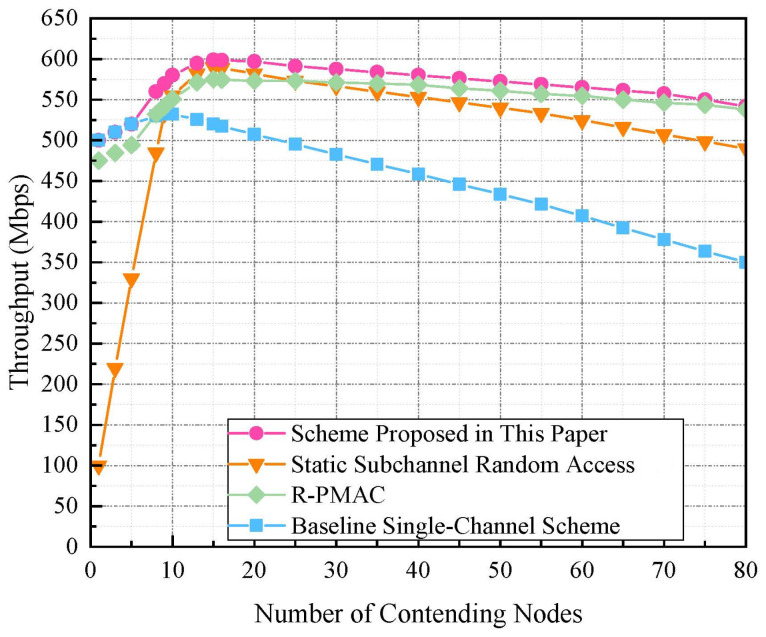
Throughput comparison of different schemes with varying node count. R-PMAC [29], Static Subchannel Random Access [34].

Figure 4 provides a comparative analysis of throughput performance across four distinct MAC mechanisms as the number of contending nodes increases. The figure demonstrates that throughput initially increases with node count but subsequently declines as node density continues to escalate, a trend primarily attributable to intensified collisions and consequent delays during the backoff process, which exacerbate network congestion. The baseline single-channel approach exhibits a pronounced decline in throughput with increasing contention, illustrating the limitations of systems without channel division in managing escalated traffic. In contrast, the algorithm proposed in this paper maintains higher and more consistent throughput levels, even as the number of nodes increases. This robust performance suggests that its adaptive channel division and subchannel allocation strategies successfully mitigate collisions, optimize bandwidth utilization, and alleviate inter-node competition. On the other hand, the random-access scheme introduced in [34] performs suboptimally at lower node densities, primarily due to its inflexible subchannel configuration. The P-PMAC algorithm from [29], despite an initial throughput disadvantage due to preamble overhead, demonstrates remarkable stability as node count escalates, indicating its robust design tailored to sustain reliable communication in noisy and high-density environments.

**Figure 5:**
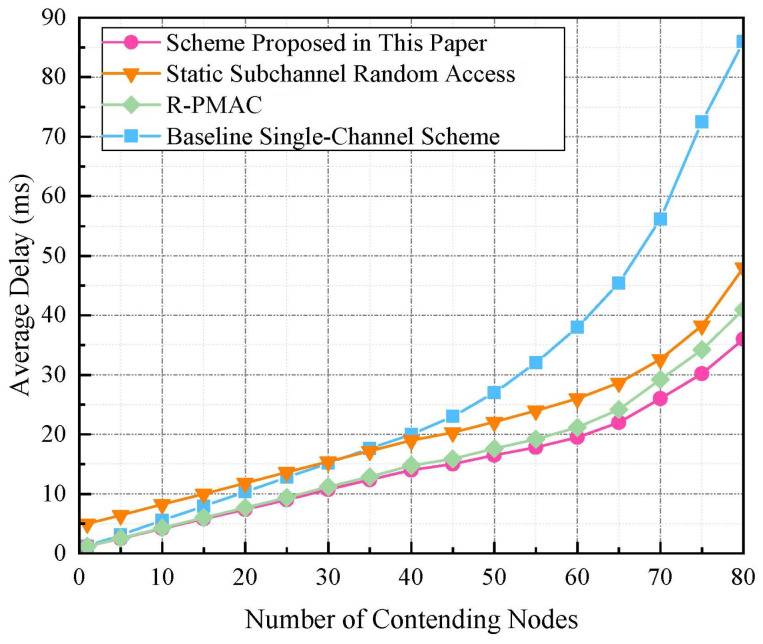
Average delay comparison of different schemes with varying node counts. R-PMAC [29], Static Subchannel Random Access [34].

Figure 5 offers a comprehensive analysis of average frame delay across four different MAC schemes as the number of contending nodes increases. Frame delay is defined as the time taken for a data frame to travel from the source node to the destination node, a critical metric in assessing the efficiency and performance of communication systems. Notably, the baseline single-channel scheme demonstrates the steepest increase in delay, indicative of its inadequate capacity in managing the increased collisions and extended backoff scenarios commonly associated with rising node density. In contrast, the static subchannel random-access scheme proposed in [34] exhibits a more moderate delay escalation, benefiting from its multiple-subchannel configuration. The R-PMAC scheme with a preamble-based collision management mechanism shows enhanced performance with a gentler slope in delay increase, reflecting its robust design in managing interference efficiently. Remarkably, the scheme proposed in this paper maintains the lowest delay across all tested scenarios, illustrating its superior adaptability through dynamic channel division and subchannel allocation.

To assess the fairness in the allocation of network resources among nodes, we utilized Jain’s fairness index [42], where the fairness index *F* is defined as

(19) $$F:=\frac{{\left(\sum_{i=1}^{N}\rho_{i}\right)}^{2}}{N\cdot \sum_{i=1}^{N}\rho_{i}^{2}}$$

where $\rho_{i}$ denotes the total throughput of node *i* during the simulation. An index value approaching one indicates a higher degree of equity within the network scheme.

**Figure 6:**
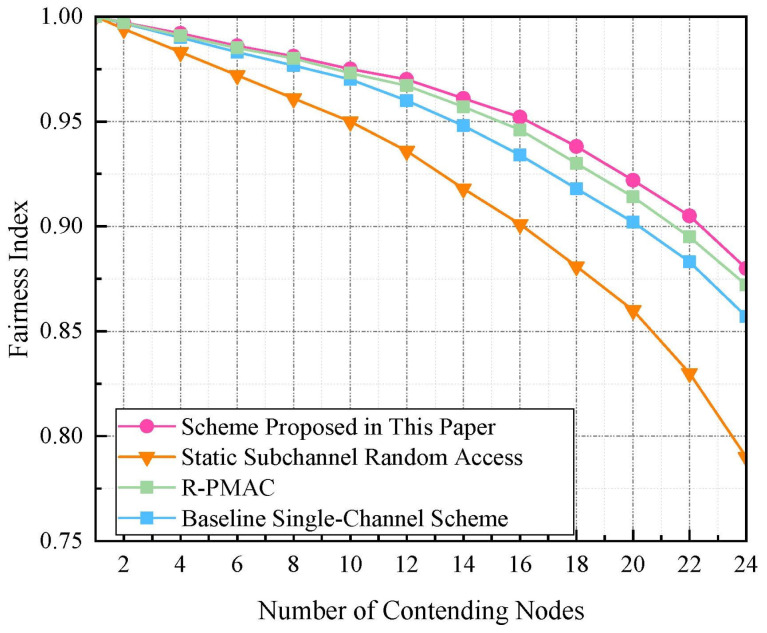
Fairness index comparison of different schemes with varying node counts. R-PMAC [29], Static Subchannel Random Access [34].

Figure 6 illustrates the variation in Jain’s fairness index for four different MAC schemes as the number of contending nodes increases. The scheme proposed in this paper maintains a relatively stable fairness index close to 1, indicating a consistent and equitable distribution of resources among nodes, even as contention increases. This stability results from the design of the subchannel allocation strategy that allows nodes to randomly determine their backoff counter value and contend for transmission opportunities equally. In contrast, the static subchannel random-access method introduced in [34] shows a gradual decrease in fairness, primarily because it favors nodes with superior channel quality, potentially leading to resource starvation for nodes in less favorable channel conditions. R-PMAC also exhibits a decline in fairness but at a slower rate, indicating that its design partially mitigates the unfair resource allocation that typically exacerbates with increasing node counts. Notably, the baseline single-channel scheme exhibits the most significant decline in fairness, illustrating the challenges of managing resource allocation without channel division, especially as the number of contending nodes grows. This stark decrease may be due to the inability of a single-channel approach to effectively handle high contention without sophisticated mechanisms to prevent collisions and manage bandwidth among a large number of nodes.

## 7. Conclusions

In conclusion, this paper addresses the critical need for more efficient communication protocols that are adaptable to the dynamic environment of airborne systems. We have developed and implemented adaptive algorithms for channel division and subchannel allocation specifically tailored to enhance the efficiency of OFDMA-based airborne PLC networks. Extensive simulation results, obtained using the OMNeT++ simulator, affirm that our algorithms surpass existing methods across several key metrics. These include throughput maximization, reduction in average delay, and equitable resource distribution among nodes—factors that are vital for maintaining operational efficiency in high-density node environments typical of airborne systems.

The proposed algorithms not only improve throughput and reduce delays but also ensure fair resource allocation across the network, thereby preserving service quality even as the number of contending nodes increases. Such performance enhancements are crucial in airborne communication scenarios where bandwidth efficiency and delay minimization are paramount. The first algorithm leverages a feedback-centric design, enabling real-time adaptability to network dynamics by adjusting the subchannel count in response to fluctuations in node count and channel conditions. Meanwhile, the second algorithm optimally integrates signal quality and current occupancy data for each subchannel, thereby preventing congestion and ensuring balanced load distribution across the network.

Both algorithms minimize the need for extensive data collection by centralizing calculations at the Central Coordinator (CCo), which significantly alleviates the computational burden on individual network devices. Collectively, these advancements present substantial promise for the future of airborne network communications, charting a course toward more reliable, fair, and efficient airborne communication systems that can meet the evolving demands of modern aviation technology.

Looking ahead, further research could explore the integration of machine learning techniques to predict network behavior more accurately and preemptively refine the allocation algorithms for even better performance. Additionally, investigating the effects of cross-layer interactions on system-wide performance could uncover opportunities to achieve further improvements, thereby enhancing the robustness and efficiency of airborne PLC networks.

## Data Availability

The data are contained within the article.

## References

[1] Coutinho Y.F., Camponogara A., Filomeno M.d.L., de Campos M.L.R., Tonello A.M., Ribeiro M.V. (2024). Two Decades of Research Progress in Resource Allocation for PLC Systems: From Core Concepts to Frontiers. IEEE Commun. Surv. Tutor..

[2] de Oliveira R.M., Vieira A.B., Latchman H.A., Ribeiro M.V. (2019). Medium Access Control Protocols for Power Line Communication: A Survey. IEEE Commun. Surv. Tutor..

[3] Beshir A.H., Negri S., Wu X., Liu X., Wan L., Spadacini G., Pignari S.A., Grassi F. (2023). Behavioral Model of G3-Powerline Communication Modems for EMI Analysis. Energies.

[4] Gonzalez-Ramos J., Uribe-Perez N., Sendin A., Gil D., de la Vega D., Fernandez I., Nunez I.J. (2022). Upgrading the Power Grid Functionalities with Broadband Power Line Communications: Basis, Applications, Current Trends and Challenges. Sensors.

[5] Pinero-Escuer P.J., Malgosa-Sanahuja J., Manzanares-Lopez P., Munoz-Gea J.P. (2014). Homeplug-AV CSMA/CA Cross-Layer Extension for QoS Improvement of Multimedia Services. IEEE Commun. Lett..

[6] Taherinejad N., Lampe L., Mirabbasi S. (2017). An Adaptive Impedance-Matching System for Vehicular Power Line Communication. IEEE Trans. Veh. Technol..

[7] Degardin V., Laly P., Lienard M., Degauque P. (2014). Investigation on power line communication in aircrafts. IET Commun..

[8] Degauque P., Stievano I.S., Pignari S.A., Degardin V., Canavero F.G., Grassi F., Canete F.J. (2015). Power-Line Communication: Channel Characterization and Modeling for Transportation Systems. IEEE Veh. Technol. Mag..

[9] Camponogara A., Oliveira T.R., Machado R., Finamore W.A., Ribeiro M.V. (2019). Measurement and Characterization of Power Lines of Aircraft Flight Test Instrumentation. IEEE Trans. Aerosp. Electron. Syst..

[10] Degardin V., Junqua I., Lienard M., Degauque P., Bertuol S. (2013). Theoretical Approach to the Feasibility of Power-Line Communication in Aircrafts. IEEE Trans. Veh. Technol..

[11] Buticchi G., Wheeler P., Boroyevich D. (2023). The More-Electric Aircraft and Beyond. Proc. IEEE.

[12] Bai T., Zhang H., Wang J., Xu C., Elkashlan M., Nallanathan A., Hanzo L. (2021). Fifty Years of Noise Modeling and Mitigation in Power-Line Communications. IEEE Commun. Surv. Tutor..

[13] Alberto Del Puerto-Flores J., Luis Naredo J., Pena-Campos F., Del-Valle-Soto C., Valdivia L.J., Parra-Michel R. (2022). Channel Characterization and SC-FDM Modulation for PLC in High-Voltage Power Lines. Future Internet.

[14] Wang B., Cao Z. (2019). A Review of Impedance Matching Techniques in Power Line Communications. Electronics.

[15] Ribeiro M.V., Filomeno M.D.L., Camponogara A., Oliveira T.R., Moreira T.F., Galli S., Poor H.V. (2024). Seamless Connectivity: The Power of Integrating Power Line and Wireless Communications. IEEE Commun. Surv. Tutor..

[16] Vlachou C., Herzen J., Thiran P. (2013). Fairness of MAC Protocols: IEEE 1901 vs. 802.11. Proceedings of the 2013 17th IEEE International Symposium on Power Line Communications and Its Applications (ISPLC).

[17] Cano C., Malone D. (2015). When Priority Resolution Goes Way Too Far: An Experimental Evaluation in PLC Networks. Proceedings of the 2015 IEEE International Conference on Communications (ICC).

[18] Ayar M., Latchman H.A. (2016). A Delay and Throughput Study of Adaptive Contention Window Based HomePlug MAC with Prioritized Traffic Classes. Proceedings of the 2016 International Symposium on Power Line Communications and Its Applications (ISPLC).

[19] Sheng Z., Tian D., Leung V.C.M., Bansal G. (2018). Delay Analysis and Time-Critical Protocol Design for In-Vehicle Power Line Communication Systems. IEEE Trans. Veh. Technol..

[20] Zhu Q., Chen Z., He X. (2019). Resource Allocation for Relay-Based OFDMA Power Line Communication System. Electronics.

[21] Berger L.T., Schwager A., Pagani P., Schneider D.M. (2015). MIMO Power Line Communications. IEEE Commun. Surv. Tutor..

[22] Chen Z., Liu Y., Liu R., Yuan J., Han D. (2019). Improved CSMA/CA Algorithm Based on Alternative Channel of Power Line and Wireless and First-Time Idle First Acquisition. IEEE Access.

[23] Bertuol S., Junqua I., Degardin V., Degauque P., Lienard M., Dunand M., Genoulaz J. Numerical Assessment of Propagation Channel Characteristics for Future Application of Power Line Communication in Aircraft. Proceedings of the 10th International Symposium on Electromagnetic Compatibility (EMC Europe).

[24] Zhang R., Wu X., Lou Y., Yan F.G., Zhou Z., Wu W., Yuen C. (2024). Channel Training-Aided Target Sensing for Terahertz Integrated Sensing and Massive MIMO Communications. IEEE Internet Things J..

[25] Sarvghadi M.A., Wan T.C. (2023). Distributed and Efficient Slot Assignment-Alignment Protocol for Resource-Constrained Wireless IoT Devices. IEEE Internet Things J..

[26] Chen Z., Zhi L., Chen P., Qi Y. (2022). An MAC Layer Algorithm Based on Power Line-Wireless Dual Media Channels and Multiplexing. China Commun..

[27] Hao S., Zhang H.Y. (2021). A Cross-Layered Theoretical Model of IEEE 1901 Power-Line Communication Networks Considering Retransmission Protocols. IEEE Access.

[28] Hao S., Zhang H. (2021). MAC Performance Analysis for Reliable Power-Line Communication Networks with ARQ Scheme. Sensors.

[29] Song K., Feng B., Wu Y., Gao Z., Zhang W. (2024). R-PMAC: A Robust Preamble-Based MAC Mechanism Applied in Industrial Internet of Things. IEEE Internet Things J..

[30] de Oliveira R.M., Vieira L.F.M., Vieira M.A.M., Vieira A.B. (2021). A dynamic network coding MAC protocol for power line communication. Telecommun. Syst..

[31] Ali K., Liu A.X., Pefkianakis I., Kim K.H. (2021). Distributed Spectrum Sharing for Enterprise Powerline Communication Networks. IEEE-ACM Trans. Netw..

[32] Qian Y., Wang Z., Li J., Zhang T., Shu F. (2017). Sub-channel assignment and link schedule for In-Home power line communication network. IET Commun..

[33] Yoon S.G., Kang D., Bahk S. (2013). Multichannel CSMA/CA Protocol for OFDMA-Based Broadband Power-Line Communications. IEEE Trans. Power Deliv..

[34] Dong R., Ouzzif M., Saoudi S. (2012). Opportunistic Random-Access Scheme Design for OFDMA-Based Indoor PLC Networks. IEEE Trans. Power Deliv..

[35] Sheng Z., Kenarsari-Anhari A., Taherinejad N., Leung V.C.M. (2016). A Multichannel Medium Access Control Protocol for Vehicular Power Line Communication Systems. IEEE Trans. Veh. Technol..

[36] Vo T.N., Amis K., Chonavel T., Siohan P. (2015). A Computationally Efficient Discrete Bit-Loading Algorithm for OFDM Systems Subject to Spectral-Compatibility Limits. IEEE Trans. Commun..

[37] Yoon S.G., Bahk S. (2011). Adaptive Rate Control and Contention Window-Size Adjustment for Power-Line Communication. IEEE Trans. Power Deliv..

[38] Jung M., Chung M., Lee T. (2005). MAC throughput analysis of HomePlug 1.0. IEEE Commun. Lett..

[39] Bautista P.A.B., Urquiza-Aguiar L.F., Cardenas L.L., Igartua M.A. (2020). Large-Scale Simulations Manager Tool for OMNeT plus plus: Expediting Simulations and Post-Processing Analysis. IEEE Access.

[40] Zimmermann M., Dostert K. (2002). A multipath model for the powerline channel. IEEE Trans. Commun..

[41] Salem A., Hamdi K.A., Alsusa E. (2017). Physical Layer Security Over Correlated Log-Normal Cooperative Power Line Communication Channels. IEEE Access.

[42] Guo C., Sheng M., Wang X., Zhang Y. (2014). Throughput Maximization with Short-Term and Long-Term Jain’s Index Constraints in Downlink OFDMA Systems. IEEE Trans. Commun..

